# Cell specific apoptosis by RLX is mediated by NFκB in human colon carcinoma HCT-116 cells

**DOI:** 10.1186/1471-2121-15-36

**Published:** 2014-10-10

**Authors:** Asif Khurshid Qazi, Aashiq Hussain, Mushtaq A Aga, Shakir Ali, Subhash Chandra Taneja, Parduman Raj Sharma, Ajit Kumar Saxena, Dilip M Mondhe, Abid Hamid

**Affiliations:** 1 Cancer Pharmacology Division, CSIR-Indian Institute of Integrative Medicine, Jammu 180001, India; 2 Bio-Organic Chemistry Division, CSIR-Indian Institute of Integrative Medicine, Jammu 180001, India; 3 Department of Biochemistry, Jamia Hamdard (Hamdard University), New Delhi 110062, India

**Keywords:** Colorectal cancer, Apoptosis, Chemotherapy, Proliferation, Migration

## Abstract

**Background:**

Resistance to chemotherapy represents a major obstacle in correcting colorectal carcinomas (CRC). Inspite of recent advances in the treatment of metastatic disease, the prognosis of the patients remains poor. RLX, a vasicinone analogue has been reported to possess potent bronchodilator, anti-asthmatic and anti-inflammatory properties. However, its anti-cancer activity is unknown.

**Results:**

Here, we report for the first time that RLX has anti-cancer property against panel of human cancer cell lines and most potent activity was found against HCT-116 cells with IC_50_ value of 12 μM and have further investigated the involvement of NFκB and caspase-3 in RLX action in CRC apoptosis. Following RLX and BEZ-235 treatment in HCT-116, we observed significant down-regulation of NFκB (1 to 0.1 fold) and up-regulation of caspase-3 (1 to 2 fold) protein expressions. Additionally, morphological studies revealed membrane blebbing, cell shrinkage, chromatin condensation and finally apoptosis in HCT-116 cells.

**Conclusions:**

Overall, these findings indicate that RLX is a potent small molecule which triggers apoptosis, and promising potential candidate to be a chemotherapeutic agent.

## Background

According to Global cancer statistics, throughout world, cases of colorectal carcinoma (CRC) switched to 1.2 million with more than 600000 deaths per year [1]. In females, CRC is the second leading commonly diagnosed and third highly mortal cancer whereas in case of males, it is the third most commonly diagnosed and fourth most common cause of mortality. Promotion from normal colonic epithelial cells into a colorectal carcinoma is a multifactorial process. There are many factors like cell proliferation, inflammation, migration and angiogenesis which play an important role in CRC development and progression [2]. Conventional combination chemotherapy regimens involving FOLFOX (5-FU with leucovorin and oxaliplatin), FOLFORI (5-FU with leucovorin and irinotecan), IFL (Irinotecan, 5-FU, leucovorin) and XELOX (Capecitabine and oxaliplatin) for the treatment of colorectal cancer [3] have limited efficacy and are associated with significant toxicity.

Colorectal cancer represents a life-threatening complication of inflammatory bowel diseases where NFκB is a natural suspect in providing a mechanistic link between inflammation and carcinogenesis. Presently, it is believed that increasing knowledge on genetic control of cellular proliferation, migration and modulation of key proteins like NFκB that are aberrant in colorectal cancer have the potential to provide an effective and improved approach for its management [4]. The molecular mechanisms underlying this process have only recently started to be clarified with biochemical and genetic studies [5]. NFκB is a key inflammatory mediator involved in initiation, progression and metastasis of CRC [6]. A variety of carcinogens and tumor promoters have shown to activate NFκB. Constitutive expression of NFκB is frequently found in tumor cells and is constitutively activated in a number of human cancers via PI3K signaling [7]. Furthermore, NFκB activation is regulated by caspase-3 which cleaves IκBα, generating a cleavage fragment that potentially acts as a constitutive inhibitor of NFκB [8]. On the contrary, down regulation of NFκB has been reported to be implicated in the HCT-116 apoptotic cell death [9]. *In vitro* treatment of HCT-116 cell lines with NVP-BEZ235, a dual pan-class I PI3K and mTOR kinase inhibitor in clinical trials has shown decrease in cell viability and *ex-vivo* analysis of tumors demonstrated a 56% decreased proliferation in CRC [10].

*Adhatoda vasica Nees* (family *Acanthaceae*), commonly known as Vasaka or Arusha is a well known herb in indigenous system of medicine for its beneficial effects. Vasicinone obtained from leaves of *Adhatoda vasica Nees* has been reported for moderate degree of bronchodilator and anti-cancer activity. In previous study, it has been shown that RLX, a vasicinone analogue has varied medicinal properties [11,12]. Here, we report for the first time that RLX has potent anti-cancer property against colon cancer HCT-116 cells. Under the tested experimental conditions, we established a differential anti-cancer effect of RLX in comparison with BEZ-235. The results demonstrated that RLX inhibits cell proliferation, decreases NFκB and increases caspase-3 expression, suppresses cell migration, causes cell membrane blebbing followed by nuclear condensation of colon cancer cell and culminate apoptosis in HCT-116 cells. These findings revealed the importance of RLX as an anti-cancer agent in treatment of colon carcinoma.

## Results

### *In vitro* screening of vasicinone analogues and BEZ-235 improves growth inhibitory effect in various human cancer cell Line

**Figure 1:**
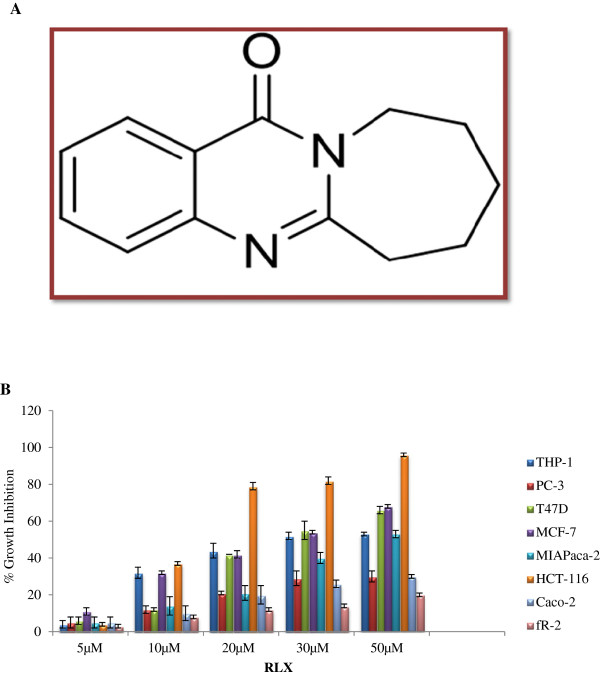
**Effect of RLX on cell proliferation. (A)** Chemical structure of RLX (7, 8, 9, 10-Tetrahydroazepino [2, 1-b] quinazolin-12(6H)-one). **(B)** Percentage growth inhibition of RLX (5 μM, 10 μM, 20 μM, 30 μM and 50 μM) against panel of human cancer cell lines including normal epithelial cell line. As depicted in Figure, RLX showed concentration dependent growth inhibition and potent effect were shown at 20 μM, 30 μM and 50 μM against HCT-116 cells.

We evaluated inhibitory efficacy *in-vitro* MTT viability assay against panel of cancer cell lines and relative IC_50_ for 48 h. Initially, we screened vasicinone analogue at indicated concentrations (5, 10, 20, 30 and 50 μM), BEZ-235 (10 nM) and 5-Flurouracil (20 μM) (Table 1) against Leukemia (THP-1), Prostate (PC-3), Breast (MCF-7, T47D), Pancreatic (MIAPaca 2), Colon (HCT-116, Caco-2) cancer cell lines and normal epithelial cells (fR-2) for 48 h. Among molecules tested, RLX (Figure 1A) showed concentration dependent inhibitory effect on cell proliferation against THP-1, T47D, MIAPaca 2, MCF-7 and HCT-116 cancer cell lines and most potent inhibition against HCT-116 whereas no significant effect on cell viability was observed in cells treated with other analogues at same concentrations. The MTT assay result revealed that HCT-116 cells treated with RLX induced growth inhibition of the order of 96%, 82% and 79% at a concentration of 50, 30 and 20 μM (Figure 1B). However, BEZ-235 (Positive control) at 10nM showed only 50%, 53% and 51% growth inhibition against T47D, MIAPaca-2 and HCT-116 whereas 5-FU (Positive control) a known anticancer agent against colon cancer, showed 56% growth inhibition against HCT-116 with cytotoxicity against normal epithelial cells (fR-2). Furthermore, IC_50_ value of RLX was calculated against panel of cell lines which was found least against HCT-116 cells (12 μM) (Table 2). Moreover, RLX showed growth inhibition of 23%, 14%, 12%, 8% and 3% at 50, 30, 20, 10 and 5 μM against fR-2 (Normal epithelial), indicated that it requires six to eight time’s higher concentration of RLX to induce 50% cell death in normal epithelial (fR-2) cell line. Interestingly, these results depicted that RLX showed high efficiency against HCT-116 as reflected by its relative IC_50_ values and no cytotoxicity against fR-2 cells.

**Table 1:** Growth inhibitory effect of vasicinone analogues, BEZ-235 and 5-Flurouracil against panel of human cancer cell lines

**Tissue type**			**Leukemia**	**Prostate**	**Breast**		**Pancreas**	**Colon**		**Epithelial (Normal)**
**Cell type**			**THP-1**	**PC-3**	**T47D**	**MCF-7**	**MIAPaca 2**	**HCT-116**	**Caco-2**	**fR-2**
**S. No**	**Structure**	**Conc (μM)**	**% Growth inhibition**							
1		50	**53 ± 2**	30 ± 3	**66 ± 2**	**68 ± 2**	**53 ± 3**	**96 ± 1**	30 ± 3	20 ± 2
		30	**52 ± 3**	29 ± 2	**55 ± 1**	**54 ± 1**	40 ± 5	**82 ± 1**	26 ± 4	14 ± 1
		20	44 ± 4	21 ± 1	42 ± 0	42 ± 2	31 ± 4	**79 ± 2**	20 ± 5	12 ± 2
		10	32 ± 2	12 ± 4	12 ± 5	32 ± 1	14 ± 3	37 ± 2	10 ± 2	8 ± 1
		5	4 ± 1	5 ± 3	6 ± 2	11 ± 1	5 ± 2	4 ± 1	5 ± 1	3 ± 2
2		50	42 ± 1	30 ± 2	49 ± 3	33 ± 3	47 ± 3	43 ± 3	10 ± 3	10 ± 2
		30	40 ± 2	23 ± 3	42 ± 2	20 ± 3	41 ± 2	27 ± 1	3 ± 2	6 ± 1
		20	33 ± 2	11 ± 2	35 ± 3	11 ± 3	39 ± 3	20 ± 1	2 ± 2	4 ± 2
		10	20 ± 1	7 ± 2	30 ± 3	1 ± 1	18 ± 2	18 ± 2	1 ± 3	1 ± 1
		5	8 ± 2	1 ± 3	10 ± 1	1 ± 1	9 ± 1	11 ± 3	1 ± 3	1 ± 2
3		50	45 ± 1	37 ± 3	30 ± 3	5 ± 1	44 ± 3	29 ± 1	10 ± 3	12 ± 1
		30	42 ± 2	29 ± 1	22 ± 2	2 ± 1	30 ± 2	29 ± 3	8 ± 2	10 ± 1
		20	6 ± 2	10 ± 2	21 ± 1	2 ± 2	15 ± 1	21 ± 2	3 ± 3	6 ± 2
		10	4 ± 1	9 ± 1	4 ± 1	1 ± 1	11 ± 3	21 ± 2	1 ± 1	2 ± 1
		5	3 ± 3	8 ± 2	1 ± 3	1 ± 1	10 ± 2	5 ± 3	1 ± 2	3 ± 1
4		50	30 ± 3	40 ± 1	20 ± 2	49 ± 2	12 ± 1	30 ± 2	10 ± 1	20 ± 1
		30	22 ± 1	27 ± 2	10 ± 1	40 ± 1	10 ± 2	23 ± 1	10 ± 2	16 ± 2
		20	21 ± 3	20 ± 1	8 ± 1	19 ± 3	6 ± 1	11 ± 3	7 ± 1	14 ± 1
		10	4 ± 1	18 ± 1	7 ± 1	10 ± 1	2 ± 1	7 ± 3	5 ± 1	11 ± 2
		5	2 ± 2	11 ± 3	2 ± 1	4 ± 2	1 ± 3	1 ± 3	2 ± 3	10 ± 1
5	BEZ-235	10nM	-	-	**50 ± 2**	-	**53 ± 4**	**51 ± 1**	-	26 ± 1
6	5-Flurouracil	20 μM	-	-	**-**	-	**-**	**62 ± 1**	-	**70 ± 2**

Bold values represent active concentration for particular cell lines.

**Table 2:** **Calculated IC**_**50 **_**values of RLX against human cancer cells and normal epithelial cells**

**Tissue**	**Cell line**	**IC**_**50 **_**(μM) (RLX)**
Leukemia	THP-1	27
Prostate	PC-3	>50
Breast	T47D	24
Breast	MCF-7	28
Pancreas	MIAPaca 2	25
Colon	HCT-116	12
Colon	Caco-2	>50
Epithelial (Normal)	fR-2	>>50

### Effect of RLX treatment on NFκB and caspase-3 protein expression in HCT-116 cell line

**Figure 2:**
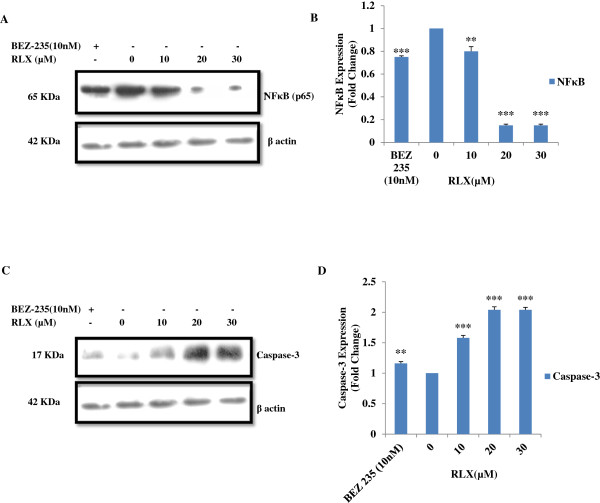
**Protein expression in presence of RLX. (A and C)** Western blot analysis revealed decrease in NFκB (p65) and increase in caspase-3 expression at 10, 20 and 30 μM of RLX and BEZ-235(10 nM) for 48 h incubation. **(B and D)** RLX treatment led significant decrease in NFκB (p65) and increase in caspase-3 expression at 30 μM with respect to untreated and BEZ-235(10 nM). ***p ≤ 0.001, **p ≤ 0.01.

We next examined the effect of RLX on NFκB and caspase-3 protein expression levels by western blotting. Following RLX (0, 10, 20 and 30 μM) treatment for 48 h, western blot analysis revealed that RLX decreased 1 to 0.1 fold expression level of NFκB (p65) (Figure 2A) and increased 1 to 2 fold caspase-3 expression level (Figure 2C). However, significant effect on expression of NFκB protein was observed at 20 and 30 μM (Figure 2B) and caspase-3 at 10, 20 and 30 μM (Figure 2D) RLX concentration as compared to untreated and BEZ-235(10nM) (Positive control) thereby suggesting NFκB down-regulation and caspase-3 up-regulation by RLX action.

### Exposure of RLX inhibits cell migration of HCT-116 cell monolayers

**Figure 3:**
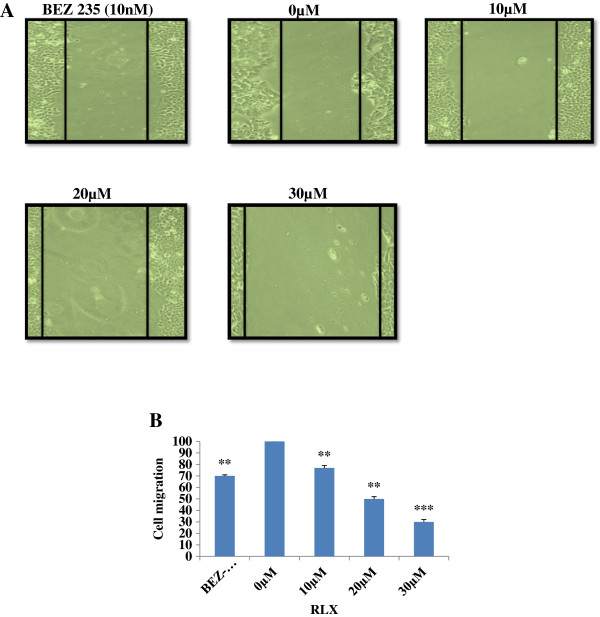
**Cell migration in HCT-116. (A)** Wound healing assay of HCT-116 stable cell lines. Cells were seeded in 6 well plate and after overnight incubation confluent monolayer cells were scraped with sterile 200 μl pipette tip and allowed to migrate for 48 h in presence of different concentrations of RLX. The wounded areas were photographed at 48 h. Wound areas were measured by Image J software. **(B)** RLX showed concentration dependent cell migration inhibition in HCT-116 monolayers. Significant inhibition on cell migration was found at 30 μM concentration of RLX as compared to untreated and BEZ-235 control. Results are presented as mean ± standard deviation. ***p ≤ 0.001, **p ≤ 0.01.

Cell migration experiment was performed to confirm the inhibitory effect of RLX in HCT-116 cells. As shown in Figure 3A, RLX treatment at 10, 20 and 30 μM showed HCT-116 cell migration inhibition when compared to untreated (0 μM) and BEZ-235 (10 nM). However, at increased concentrations of RLX (20 and 30 μM), HCT-116 cells showed significant cell migration inhibition in a concentration dependent manner as compared to untreated control (Figure 3B).

### RLX treatment culminate microvilli loss in HCT-116 cells

**Figure 4:**
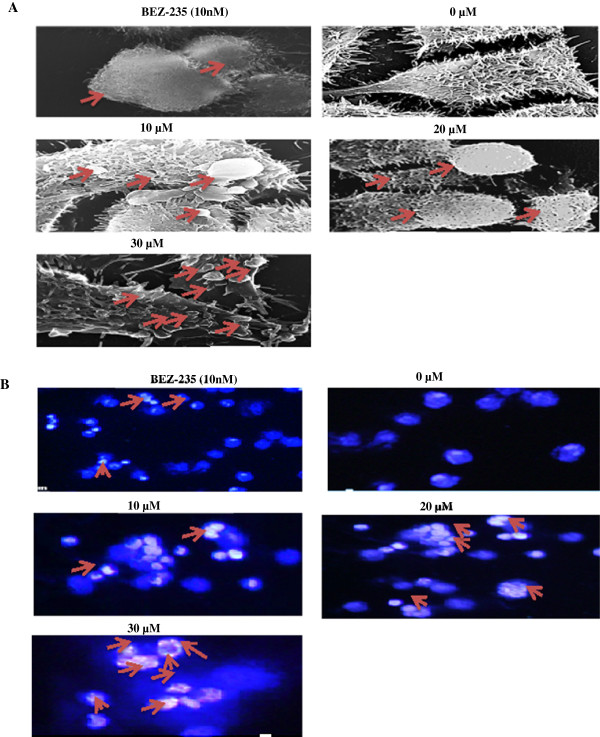
**Microscopic studies in presence of RLX. (A)** Scanning electron microscopy in HCT-116 cells. The untreated control cells (0 μM) showed rough surface and microvilli. The treatment of RLX for 24 h caused reduction in cell size, smoothening of cell surface, blebbing of the plasma membrane and loss of microvilli. **(B)** Fluorescence microscopy in HCT-116 cells. HCT-116 cells were treated with different concentration of RLX for 48 h and stained with DAPI followed by analysis for nuclear condensation. Data are representative of one of the three independent experiments.

As exhibited in Figure 4A, untreated cells were having intact microvilli all over the surface. However, RLX treatment at 10, 20 and 30 μM resulted in blebbing of the plasma membrane and loss of microvilli. Interestingly, most significant smoothening of the cell surface, shrinkage of size and blebbing of the plasma membrane and apoptotic body formation were observed at 30 μM concentration of RLX when compared with untreated and BEZ-235. Overall, SEM data clearly demonstrated typical early apoptotic phenomena and loss of microvilli as compared to untreated control.

### Nuclear condensation in RLX treated HCT-116 cells

We next examined the morphological insights caused by RLX in HCT-116 cells using diamidine phenyl indoledihrdrochloride (DAPI) staining. Uniformly spherical HCT-116 cell with normal morphology was observed in untreated cultures, whereas chromatin condensation and chromosomal DNA cleavage were observed when HCT-116 cells were treated with RLX (10, 20, 30 μM) and BEZ-235 (10 nM). As shown in Figure 4B, with increase in concentration (10, 20, 30 μM) of RLX there is increase in nuclear condensation and formation of apoptotic vesicles. However, prominent effect was seen at a concentration of 20 and 30 μM treated cultures. Overall these results suggested the ability of RLX to induce apoptotic morphology in HCT-116 cells.

## Discussions

In current study, an interesting correlation was revealed for the first time between various regulatory and phenotypic events of RLX with apoptosis. We first evaluated the growth inhibitory and cytotoxic effect of vasicinone analogues including RLX against panel of human cancer cell lines *in vitro* which includes Leukemia (THP-1), Prostate (PC-3), Breast (MCF-7, T47D), Pancreatic (MIAPaca 2), Colon (HCT-116, Caco-2) and normal epithelial cells (fR-2). Notably, we found for the first time that RLX inhibited cell growth and more importantly showed concentration dependent inhibition against panel of cancer cell lines tested. Besides, maximum and potent growth inhibition following RLX treatment was observed in human colon cancer cell line i.e. HCT-116. Keeping this in view, we further evaluated IC_50_ value of RLX against THP-1, PC-3, MCF, T47D, MIAPaca-2, HCT-116, Caco-2 and fR-2 cell lines by MTT assay, which was found minimum in HCT-116 cells. Overall, these results depicted that RLX showed significant effect against HCT-116 and least effect on Caco-2 colon cancer cell proliferation as reflected by relative IC_50_ value. Since the NFκB pathway is important for cell survival, proliferation, cell cycle progression and migration which therefore affects regulation of proliferative, anti-apoptotic, pro-apoptotic and cell cycle regulatory molecules and thus results in cell survival, proliferation, progression and migration of numerous cancers [13]. NFκB promotes cell survival via the induction of proteins that inhibits the components of apoptotic machinery in normal as well as cancerous cells [14]. To evaluate the mechanism by which effect of RLX occurred, we further examined the effect on NFκB protein expression. The most abundant form of NFκB consists of a p50 subunit and a p65 subunit. In its inactive form, NFκB is located in the cytoplasm however, upon activation by various stimuli, it translocates to the nucleus, where it may activate genes leading to cell survival or proliferation [15]. Notably, our study demonstrated that exposure to RLX resulted in remarkable down-regulation in expression of NFκB (p65). Furthermore, caspases play a pivotal role in the mechanism of apoptosis as they are both the initiators and executioners. Among caspases, caspase-3 is a frequently activated death protease, which catalyzes the specific cleavage of many key cellular proteins resulting in apoptosis [16]. Importantly, caspase-3 is crucial for apoptotic chromatin condensation and DNA fragmentation in all cell types. Here, we show that RLX mediates caspase-3 up-regulation in HCT-116 cells. Taken together, these data indicated that NFκB and caspase-3 play a pivotal role in mediating RLX induced apoptosis in HCT-116 cells. Cell migration plays a critical role in tumor cell invasion and metastasis [17]. Cell migration and invasion represents an important property for chemotherapeutic agent other than having potential to cause specific cancer cell death. Molecules involved in cancer cell migration could be potential target for anti-metastasis therapy. We summarize how colon cancer cell migrate using RLX which is tested by measuring the gap between control and treatment groups. Although RLX was found to actively inhibit colon cancer cell migration. Notably, apoptosis is morphologically characterized by chromatin condensation, inter-nucleosome fragments, cell shrinkage, membrane blebbing and formation of apoptotic bodies without disruption of plasma membrane [18]. In view of promising potential of RLX as an apoptotic agent, we further performed scanning electron microscopy (SEM) to assess early apoptotic morphological changes in HCT-116 cells. Data depicted a loss of microvilli on the surface of treated colon malignant cells and therefore revealed smoothening of cell surface which altogether validates formation of typical apoptotic feature on RLX treatment. This was supported by fluorescence microscopy using DAPI with subsequent features of cell shrinkage, membrane blebbing, chromatin condensation and nuclear fragmentation in contrast to the control cells, which retained their polygonal structure.

## Conclusions

We report for the first time that RLX has target based anticancer activity. A very important property for a candidate anti-cancer drug is the ability to induce tumor cell apoptosis [19] and RLX exhibits this important characteristic feature. These findings should be useful for development of molecule(s) targeted against various cancer signaling pathways.

## Methods

### Chemicals and source of antibodies and kits

Growth medium (MEM/RPMI), fetal calf serum, trypsin, penicillin, streptomycin, DMSO, proteinase K, RIPA Buffer, bisacrylamide, SDS, MTT dye, acrylamide, ammonium persulfate (APS), N, N, N’, N’ tetramethylethylenediamine (TEMED), 2-mercaptoethanol, DAPI, Tris base. All the above mentioned chemicals were obtained from Sigma. Chemiluminescent western blotting kit (Millipore), Quanti Pro BCA assay kit, 96 and 6 well plate (Iwaki), triton X (Hi-Media), EDTA (Hi-Media), ELISA plate reader (Bio-Rad). NFκB (p65), caspase-3 antibodies were purchased from Millipore Pvt Ltd.

### Synthesis and structure of RLX

Synthesis of RLX been reported previously [11]. Chemical structure of RLX is shown in Figure 1A.

### Cell lines, growth medium and treatment conditions

Human cancer cell lines; Leukemia (THP-1), Prostate (PC-3), Colon (HCT-116, Caco-2), Breast (T47D, MCF-7) Pancreatic (MIAPaca 2) and Normal epithelial (fR-2) were procured from European Collection of cell culture (ECACC), UK. Cells were grown in Minimum Essential Medium (MEM) and Roswell Park Memorial Institute medium (RPMI) supplemented with 10% FCS and 1% penicillin. Penicillin was dissolved in PBS and sterilized by filtering through 0.2 μm filter in laminar air flow hood. Cells were cultured in CO_2_ incubator (New Brunswick, Galaxy 170R, eppendroff) with an internal atmosphere of 95% air and 5% CO_2_ gas and the cell lines were maintained at 37°C. The media was stored at low temperature (2-8°C) and the medium for cryopreservation contained 20% FCS and 10% DMSO in growth medium.

### Cell viability assay

The MTT assay was used to assess the effect of the molecules on cell viability. In each well of a 96-well plate, cells with different densities were grown in 100 μL of medium. After 24 h, RLX was added to achieve a final concentration of 50, 30, 20, 10, 5 μM, BEZ-235 (10 nM) and 5-Flurouracil (20 μM) (Positive controls) respectively. 4 h prior to the completion of 48 h treatment of RLX and BEZ-235, 20 μL of 2.5 mg/mL of MTT solution in PBS was added to each well. After 48 h, supernatant was removed and formazan crystals were dissolved in 150 μL of DMSO. Absorbance was then measured at 570 nm using an absorbance plate reader (Bio-Rad Microplate Reader). Data was expressed as percentage of the viable cells in treated relative to untreated conditions. The experiments were repeated thrice and carried in triplicates [20].

### Preparation of whole cell lysates and western blot analysis

HCT-116 (2 × 10^6^ cells/ml/well) cells were treated with RLX at 0 (untreated), 10, 20, 30 μM and BEZ-235 (10 nM) (Positive control) for 48 h. After that cells were trypsinized and suspended in cold RIPA buffer (150 mM NaCl, 1.0% IGEPAL CA-630, 0.5% sodium deoxycholate, 0.1% SDS, 50 mM Tris, PH 8.0) for 30 min on ice. The lysates were vortexed and then centrifuged at 14,000 g for 15 min. Supernatant thus obtained was whole cell lysate which was stored at -20°C for future use. Protein content was measured using BSA (1 mg/ml) and samples with unknown concentrations were plotted in a linear range of 0.5 to 30 μg/ml of the protein concentration and absorbance measured at 562 nm. The above protein lysates were subjected to discontinuous SDS-PAGE at 100 V and electro transferred to polyvinylidene difluoride (PVDF) membrane (Millipore) for 2 h at 120 V at 4°C. The membrane was blocked with 3% skimmed milk in PBS for 1 h. After blocking, the membrane was probed with specific primary antibody for overnight at 4°C followed by 3 times washing with TBST for 5 min each. A dilution of secondary antibody (mouse and rabbit) conjugate was added for 1 h of incubation and signals were detected using Millipore Chemiluminescent western blotting kit and analyzed using X-ray film [20].

### Cell migration Assay

HCT-116 cell monolayer (90% confluent) was allowed to become quiescent in medium with 0.1% dialyzed fetal bovine serum for 24 h. Further, cells were scraped to make a straight line wound and treated with RLX and BEZ-235 (Positive control) for 48 h. Photographs were taken through an inverted microscope (×40 magnification) at 48 h and lengths of wound were determined by Image J (version 1.46) software [21].

### Cell surface examination with scanned electron microscopy

HCT-116 (0.5 × 10^5^ cells/ml) were treated with RLX at 0 (untreated), 10, 20, 30 μM and BEZ-235(10nM) (Positive control) concentration for 24 h. Following PBS wash, fixation of cells was done with 2.5% glutaraldehyde in 0.1 M cacodylate buffer for 1 h at 4°C and post fixed with 1% OsO_4_ in the same buffer for 1 h at room temperature. Dehydration was done with ascending grades of acetone following critical point drying using liquid CO_2._ Gold coating (thickness 20 nm) was done using sputter coater and viewed under electron microscope (JEOL JEM -100CXII) with ASID at 40KV [20].

### Apoptotic characterization using fluorescence microscopy

HCT-116 **(**5 × 10^5^ cells/ml/well) cells were treated with RLX 0 (untreated), 10, 20, 30 μM and BEZ-235 (10 nM) (Positive control) concentrations. After 48 h of incubation, cells were centrifuged at 3000 rpm for 5 min. Resuspended pellet was dissolved in PBS. The air dried smears were fixed in methanol at -20°C, stained with DAPI (1 μg/ml) and kept at 37°C for 20 minutes. Following PBS wash, mounting was done with glycerol: PBS (90:10) on coverslip and prepared slides were observed under fluorescence microscope (Olympus) using UV filter at 40× magnification [20].

### Statistical evaluation

The results of three independent experiments were expressed as the mean ± SD. Statistical evaluation was performed using an un-paired t-test. ***p ≤ 0.001, **p ≤ 0.01, *p ≤ 0.05.

## Competing interests

The authors declare that they have no competing interests.

## Authors’ contributions

AKQ carried out cellular studies. AH helped to draft the manuscript. MAA performed chemisty studies. SA helped in data analysis and interpretation. SCT participated in data analysis and interpretation of chemisty part. PRS carried out microscopic studies. AKS performed manuscript review. DMM helped in data analysis and interpretation. AH participated in its design and interpretation. All authors read and approved the final manuscript.
